# Baseline and early ^18^F-FDG PET/CT evaluations as predictors of progression-free survival in metastatic breast cancer patients treated with targeted anti-CDK therapy

**DOI:** 10.1186/s40644-024-00727-2

**Published:** 2024-07-09

**Authors:** Charline Lasnon, Adeline Morel, Nicolas Aide, Angélique Da Silva, George Emile

**Affiliations:** 1 Nuclear Medicine Department, François Baclesse Comprehensive Cancer Center, UNICANCER, 3 Avenue du General Harris, BP 45026, Caen Cedex 5, 14076 France; 2 UNICAEN, INSERM 1086 ANTICIPE, Normandy University, Caen, France; 3 Medical Oncology Department, François Baclesse Comprehensive Cancer Center, UNICANCER, Caen, France

**Keywords:** Breast cancer, Targeted therapy, Cyclin-dependent kinase inhibitor, FDG, PET

## Abstract

**Background:**

Exploring the value of baseline and early ^18^F-FDG PET/CT evaluations in prediction PFS in ER+/HER2- metastatic breast cancer patients treated with a cyclin-dependent kinase inhibitor in combination with an endocrine therapy.

**Methods:**

Sixty-six consecutive breast cancer patients who underwent a pre-therapeutic ^18^F-FDG PET/CT and a second PET/CT within the first 6 months of treatment were retrospectively included. Metabolic tumour volume (MTV) and total lesion glycolysis (TLG) and D_max_, which represents tumour dissemination and is defined as the distance between the two most distant lesions, were computed. The variation in these parameters between baseline and early evaluation PET as well as therapeutic evaluation using PERCIST were assessed as prognosticators of PFS at 18 months.

**Results:**

The median follow-up was equal to 22.5 months. Thirty progressions occurred (45.4%). The average time to event was 17.8 ± 10.4 months. At baseline, D_max_ was the only predictive metabolic parameter. Patients with a baseline D_max_ ≤ 18.10 cm had a significantly better 18 m-PFS survival than the others: 69.2% (7.7%) versus 36.7% (8.8%), *p* = 0.017. There was no association between PERCIST evaluation and 18 m-PFS status (*p* = 0.149) and there was no difference in 18 m-PFS status between patients classified as complete, partial metabolic responders or having stable metabolic disease.

**Conclusion:**

Disease spread at baseline PET, as assessed by D_max_, is predictive of an event occurring within 18 months. In the absence of early metabolic progression, which occurs in 15% of patients, treatment should be continued regardless of the quality of the initial response to treatment.

## Background

Breast cancer (BC) is the most common malignancy in women in the world and currently accounts for 1 in 8 cancer diagnoses and a total of 2.3 million new cases for both sexes [1]. Around 6–10% of BC are diagnosed with *de novo* metastatic disease and 25–30% present a metastatic relapse [2]. Metastatic breast cancer (mBC) has a poor survival with a 5-year relative survival rate of 38% in Europe [3]. BC is a heterogeneous disease with different clinical and molecular characteristics. Approximately 80% of BC are estrogen receptor-positive (ER+), and most patients therefore benefit from endocrine therapy (ET) [4]. ET is the main treatment for patients with ER+/HER2- (human epidermal growth factor receptor 2 negative) mBC. Nevertheless, resistance to ET often develops, requiring a switch to another treatment to escape the resistance mechanisms.

The advent of cyclin-dependent kinase inhibitors (CdK4/6i) has considerably improved the prognosis of mBC. Palbociclib, ribociclib and abemaciclib, recently approved by the FDA (Food and Drug Administration) and EMA (European Medicines Agency), are now the gold standard for first-line treatment of ER+/HER2- mBC without extensive visceral involvement [3]. CdK4/6i prevent DNA replication by blocking progression from the G1 to S phase during cell division and tumor cell proliferation [5, 6], meaning that they arrest tumor growth rather than causing significant tumor cell death. Recently, preclinical studies demonstrated that palbociclib improves the efficacy of cytostatic agents such as ET with synergistic effects [7, 8]. These new targeted therapies require new imaging tools to assess treatment response [9].

^18^F-FDG PET/CT is increasingly used to monitor therapeutic response in several cancers treated with new targeted therapies [10]. In patients followed for BC, ^18^F-FDG PET/CT has a role in staging locally advanced disease and assessing response to chemotherapy and ET [11, 12]. In the quest to develop personalized medicine, ^18^F-FDG PET/CT could also be a useful imaging tool for evaluating the response of BC to new targeted therapies, helping clinicians to select patients for whom it is indicated to continue treatment and those for whom, conversely, it is indicated to discontinue or modify the therapeutic management. To our knowledge, ^18^F-FDG PET/CT for the therapeutic evaluation of CdK4/6i in the context of BC has received very little attention [13–15] and only in small series of patients. The most recent study suggested that early metabolic changes assessed by ^18^F-FDG-PET after the initiation of CdK4/6i might identify patients at risk of treatment failure [16].

We therefore explored the value of baseline and early ^18^F-FDG PET/CT evaluations in predicting progression free survival (PFS) in a larger consecutive series of ER+ / HER2- mBC patients treated with CdK4/6i in a specialist breast center certified by the European Society of Breast Cancer Specialists (EUSOMA) over the period of inclusion [17].

## Methods

### Population

All patients with a histologically proven ER+ / HER2- mBC treated with an CdK4/6i in combination with an ET as first or second line of treatment, and who underwent a pre-therapeutic ^18^F-FDG PET/CT between January 2018 and December 2020 and a second ^18^F-FDG PET/CT within the first 6 months of treatment, were retrospectively included. The age as well as the type of CdK4/6i and ET were recorded from the patients’ medical records. All patients were followed up for a minimum period of 18 months to assess the 18-month progression-free survival (18m-PFS). The endpoint was defined as the time from the introduction of CdK4/6i until relapse or progression. Events were assessed according to the conclusions of the multidisciplinary tumor board.

All procedures performed in studies involving human participants were approved by the local ethics committee and were in accordance with the 1964 Helsinki Declaration. In accordance with European regulations, observational studies without any additional therapy or monitoring procedures do not need the approval of an ethical committee. Additionally, the need for informed signed consent was waived. The procedure was declared to the National Institute for Health Data, under registration no. F20220902111241.

#### PET examinations

All PET examinations were performed either on a TrueV Biograph PET/CT system (Siemens Healthineers, USA) or a VEREOS PET/CT system (Philips Medical Solutions, USA) according to the European Association of Nuclear Medicine procedure guidelines for tumor imaging [18]. Patients who had been fasting for at least 4 h were injected with 3MBq/kg of ^18^F-FDG and scanned from skull base to mid-thighs on the PET devices with the following parameters, both systems being EARL-accredited:

- On the TrueV analogic PET/CT, PET was performed for 2 min and 40 s and 3 min and 40 s per bed position for normal-weight and overweight patients, respectively. Images were reconstructed using three iterations and 21 subsets with point spread function (PSF) reconstruction.
- On the Vereos digital PET/CT, PET was performed from the skull to mid-thighs for 2 min per bed position, regardless of the body habitus of the patients. Images were reconstructed using two iterations and 10 subsets with PSF reconstruction.

### PET analysis

All PET examinations were analyzed using Syngo.via (Siemens Healthineers, USA) and LifeX Software (version 7.4.0). The EARL standardization strategy was used to ensure consistency of quantitative values between the two PET devices [19]. According to PERCIST criteria [20], only lesions with SUV_peak_ of at least 1.5-fold greater than liver SUV_mean_ + 2 SDs (in 3-cm spherical VOI in normal right lobe of liver) were considered and were segmented using an adaptive threshold method. The total metabolic volume (MTV) was calculated by summing the MTV of each lesion. The lesion glycolysis of a lesion was the product of MTV and the mean SUV of each lesion. The total lesion glycolysis was computed by summing the TLG of each lesion. D_max_ was also calculated on each examination. It is a way to represent tumor dissemination and to refine tumor staging [21]. It is defined as the distance (in cm) between the two lesions that are the furthest apart (with VOIs center of mass as origin).

For the PERCIST one-lesion analysis, the SUL_peak_ of the hottest lesion per patient was used. Of note, the hottest lesion could be different between the two PET examinations. ΔSUL_peak_ was computed as follows:

$${\Delta }{SUL}_{peak}= \frac{{SUL}_{peak }2- {SUL}_{peak} 1}{{SUL}_{peak} 1}*100$$

The same equation was used to compute ΔMTV and ΔTLG.

According to PERCIST criteria, the patient was considered:

- in complete metabolic response (CMR) if all lesions had disappeared.
- in partial metabolic response (PMR) if the SUV_peak_ of the hottest lesion was reduced by more than 30%.
- in progressive metabolic disease (PMD) if the SUV_peak_ of the hottest lesion increased by more than 30% or in the event of a new lesion(s).
- in stable metabolic disease (SMD) otherwise.

### Statistical analysis

Quantitative data are presented as mean ± standard deviation, otherwise specified. For PFS, the endpoint was defined as the time from the introduction of the treatment until relapse, progression or death as a result of BC. Baseline clinical and PET characteristics of all patients with no events at 18 months (18 m-PFS0) and patients who experienced an event at 18 months (18 m-PFS1) were compared using chi-square tests or Mann-Whitney tests as appropriate. Spearman analyses were used to seek correlations between baseline quantitative PET parameters.

At early PET evaluation, as per clinical practice and expected results of such cytostatic treatment, CMR, PMR and SMD patients were considered as responder patients, whereas PMD patients were considered as non-responder (NR) patients. Clinical and PET characteristics of the three classes of responders were compared using chi-square tests or Mann-Whitney tests as appropriate.

A receiver operator characteristic curve was generated to calculate the area under the curve (AUC) to determine the optimal cut-off value of quantitative PET parameters of interest for predicting 18 m-PFS using the Youden index method. Eighteen-month PFS curves were plotted using Kaplan-Meier survival analyses with log-rank tests. Univariate Cox regression models were used to calculated hazard ratios (HR) and their corresponding 95% confidence interval. Statistical analysis was performed using XLSTAT (version 2020.1.1). A *p* value of less than 0.05 was considered statistically significant.

## Results

### Population characteristics

Seventy mBC patients were screened. Three patients were excluded due to non-hypermetabolic disease and one patient was excluded due to non-adherence to treatment. Clinical characteristics of the 66 included patients can be found in Table 1.

**Table 1:** Patient characteristics

Variables	Data
**Age (years)**, mean (range)	60 (32–93)
**Histological type**, n (%)	
Invasive ductal carcinoma	59 (89.4)
Invasive lobular carcinoma	7 (10.6)
**Number of lesions**, n (%)	
≤ 5	49 (74.2)
**Number of lesions**, n (%)	
≤ 5	49 (74.2)
>5	17 (25.8)
**Tumoral sites**, n (%)	
Bone	48 (72.7)
Lymph nodes	40 (60.6)
Pleuropulmonary	19 (28.8)
Breast or Parietal	17 (25.7)
Liver	16 (24.2)
Others *	8 (12.1)
**Line of metastatic treatment**, n (%)	
First line	58 (87.9)
Second line	8 (12.1)
**CDK4/6 inhibitor combination**, n (%)	
AI + Abemaciclib	8 (12.1)
Fulvestrant + Abemaciclib	4 (6.1)
AI + Palbociclib	33 (50.0)
Fulvestrant + Palbociclib	6 (9.1)
AI + Ribociclib	15 (22.7)
**Goserelin**, n (%)	18 (26.9)

AI: aromatase inhibitor, *peritoneum, adrenals and soft tissues

The mean delay between the baseline PET examinations and the introduction of CdK4/6i was equal to 39 ± 30 days. Early PET evaluation was done on average 2.8 (± 1.0) months after the introduction of treatment. Of the 11 patients who commenced treatment more than 2 months following the baseline PET scan, 5 underwent local palliative/antalgic radiotherapy, while 5 underwent histological confirmation; the latest data was unavailable.

The median follow-up was equal to 22.5 months, during which 30 progressions occurred (45.4%). Out of these 30 patients who experienced an event, 7 exhibited multisite progression. Specifically, 17 patients showed bone progression, 10 hepatic, 5 nodal, 3 pulmonary, 2 peritoneal, 1 pleural and 1 parietal progression. All were authenticated through PET imaging, with 14 additionally confirmed by clinical symptomatology, tumor biomarkers, or targeted radiological imaging focusing on suspected areas of progression. The average time to occurrence of an event was 17.8 ± 10.4 months (min: 1.0, max: 18.0). Five deaths were recorded within 18 months of treatment initiation, all occurring after progression was observed.

### Baseline PET predictive value

Baseline PET characteristics of patients according to their 18 m-PFS status are displayed in Table 2.

**Table 2:** Baseline PET characteristics for entire population according to 18-month PFS status

Parameters	Whole population (*n* = 66)	18 m-PFS0 (*n* = 36)	18 m-PSF1 (*n* = 30)	*P* value
**SUV**_**peak**_, mean (SD)	6.69 (2.61)	6.78 (2.45)	7.19(2.81)	0.580
**TLG**, mean (SD)	486.68 (1946.63)	391.14 (1757.94)	601.33 (2176.87)	0.081
**MTV**, mean (SD)	93.73 (355.76)	75.50 (333.34)	115.60 (385.58)	0.081
**D**_**max**_, mean (SD)	21.20 (20.31)	15.65 (16.81)	27.86 (22.34)	0.025
**Number of lesions**, mean (SD)	5.50 (10.02)	4.19 (4.64)	7.07 (13.94)	0.184
**Metastatic locations**, n (%)				0.199
Only bone	17 (25.8)	7 (19.4)	10 (33.3)	
Multiple locations	49 (74.2)	29 (80.6)	20 (66.7)	

SUV: Standardize Uptake Value, TLG: Total Lesion Glycolysis, MTV: Metabolic Tumor Volume, D_max_: Maximum distance between two lesions

At baseline, D_max_ was significantly higher in 18 m-PFS1 patients than in 18 m-PFS0 patients, with mean (SD) D_max_ equal to 27.86 cm (22.34) and 15.65 cm (16.81), respectively. Baseline SUV_peak_, MTV, TLG, number of lesions and metastatic locations were not different between 18 m-PFS0 and 18 m PSF1 patients. Additionally, there was no difference in D_max_ between patients with solely bone metastatic disease and those with multiple metastatic sites: 16.88 cm (19.02) versus 22.70 cm (20.71), respectively (*p* = 0.193).

**Fig. 1:**
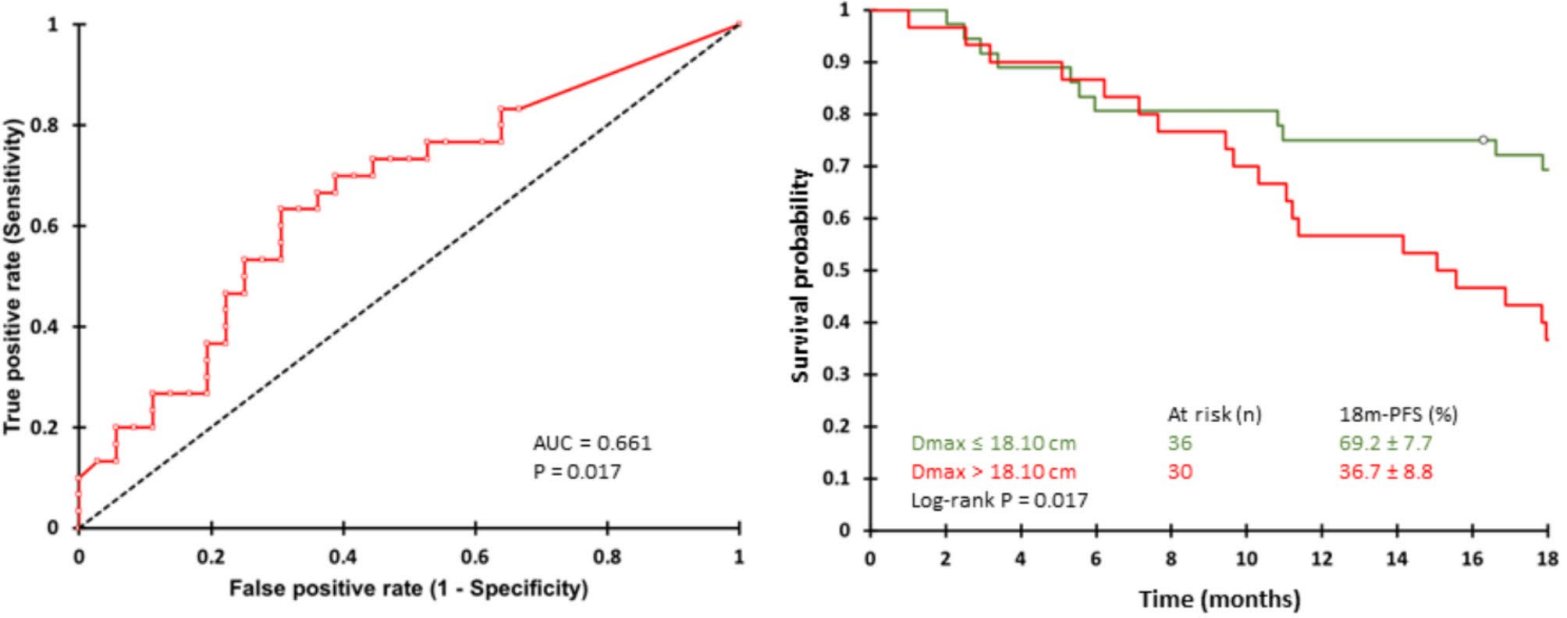
ROC curves and Kaplan-Meier analyses of baseline D_max_ for all patients

A ROC curve analysis of D_max_ according to 18 m-PFS status displayed an area under the curve of 0.661 (95%IC = 0.528–0.793, *p* = 0.01). It determined that the optimal baseline D_max_ threshold to discriminate between 18 m- PFS0 and 18 m-PFS1 patients was 18.10 cm. Kaplan-Meier analysis showed that patients with a baseline D_max_ ≤ 18.10 cm had a significantly better 18 m-PFS survival than the others: 69.2% (7.7%) versus 36.7% (8.8%), *p* = 0.017 (Fig. 1).

**Fig. 2:**
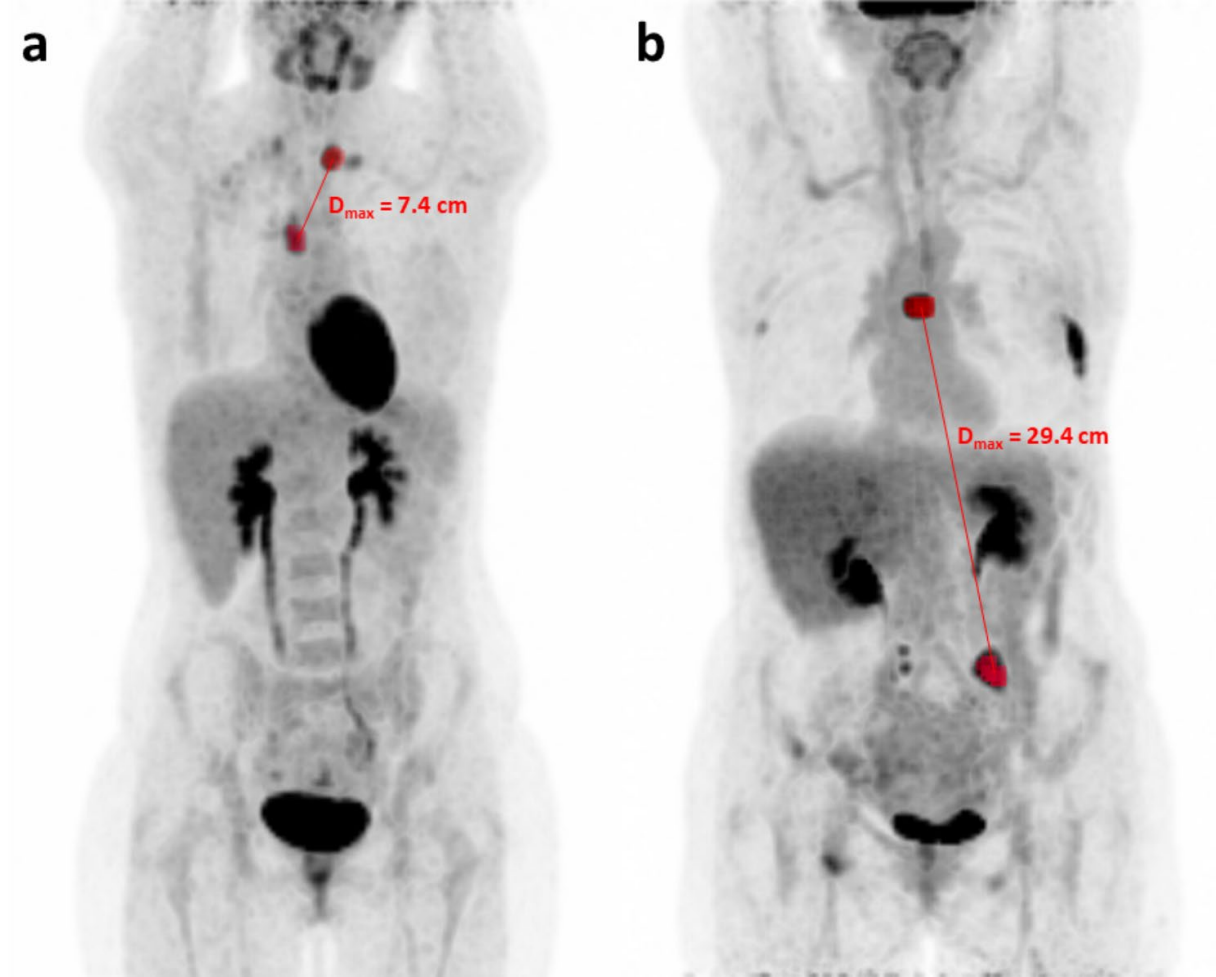
Representative example of patients with favorable and unfavorable baseline D_max_. **(a)** A 32-year-old patient in first-line metastatic treatment (Letrozole – Ribociclib - Goserelin) with a baseline D_max_ of 7.4 cm and no event within 18 months of treatment initiation. **(b)** A 54-year-old patient in first-line metastatic treatment (Letrozole – Palbociclib) with a baseline D_max_ of 29.4 cm who had an early metabolic progression at 3.2 months after initiation of treatment. The most distant lesions are highlighted in red in each case

A representative example of these two categories of patients is displayed in Fig. 2.

Finally, a univariable Cox proportional hazards model indicated a 1.02-fold increase in risk of progression for each additional centimeter of D_max_ at baseline PET evaluation: HR = 1.018 (95%IC = 1.002–1.034), *p* = 0.031.

### Early evaluation PET predictive value

**Fig. 3:**
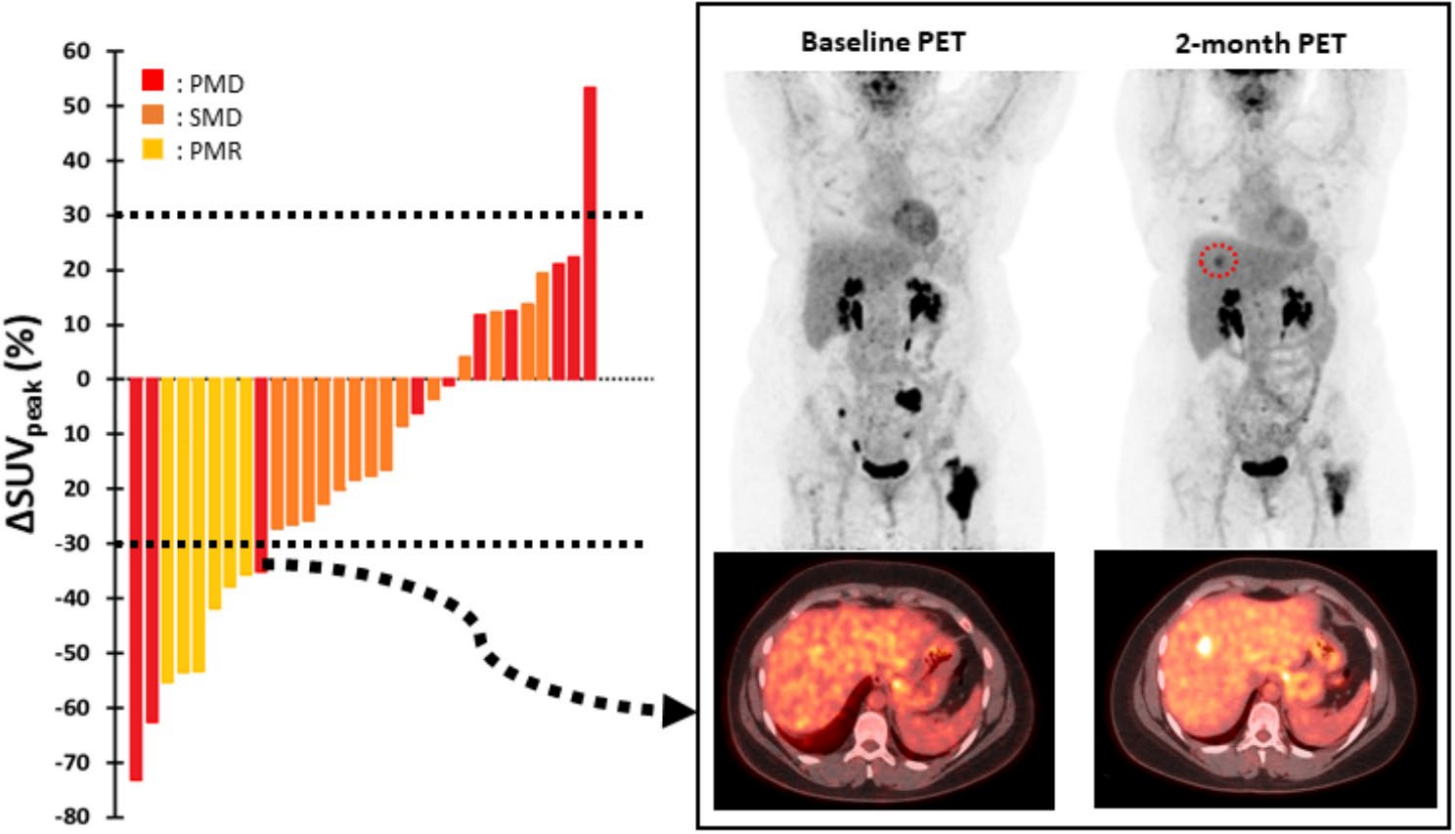
Metabolic characteristics of patients in non-complete metabolic response. On the left panel, waterfall plot of ΔSUVpeak shows that only one patient classified as PMD had more than a 30% increase in SUVpeak, with all having new lesions. Of note, three of these PMD patients had new lesions, even though a significant decrease in SUVpeak was observed. The right panel displays a representative case of one of these patients

Early PET evaluation allowed the identification of 10 early progressions (15.1%). The other 20 progressions occurred later. All patients classified as early PMD presented with new lesions but only one had an increase of more than 30% in SUV_peak_. Of note, three of these PMD patients had new lesions, even though a significant decrease in SUV_peak_ was observed (Fig. 3).

Early progressors had mean baseline SUV_peak_, MTV, TLG and D_max_ equal to 7.56 ± 3.03, 32.2 ± 31.42, 174.4 ± 237.57 mL, and 17.51 ± 15.65 cm, respectively. Of the 56 responder patients, 36 were in CMR, 6 in PMR and 14 in SMD. A waterfall plot with the ΔSUV_peak_ between early and baseline PET/CT examinations for non-complete responder patients can be found in Fig. 3. In responder patients, the decreases in SUV_peak_, MTV, TLG and D_max_ were not different between 18 m-PFS0 and 18 m-PFS1 patients (Table 3).

**Table 3:** Early PET characteristics of responder patients according to 18-month PFS status

Parameters	Whole population (*n* = 56)	18 m-PFS0 (*n* = 36)	18 m-PFS1 (*n* = 20)	*P* value
**ΔSUV**_**peak**_, mean (SD)	-71.70 (40.46)	-79.63 (34.74)	-57.44 (46.71)	0.059
**ΔTLG**, mean (SD)	-89.86 (25.56)	-90.18 (22.60)	-84.95 (32.56)	0.137
**ΔMTV**, mean (SD)	-90.39 (24.48)	-89.59 (25.38)	-85.57 (30.90)	0.172
**ΔD**_**max**_, mean (SD)	-85.10 (38.61)	-88.45 (39.06)	-79.07 (38.01)	0.185
**PERCIST response**, n (%)				0.149
CMR	36 (64.3)	26 (72.2)	10 (50.0)	
PMR	6 (10.7)	4 (11.1)	2 (10.0)	
SMD	14 (25.0)	6 (16.7)	8 (40.0)	

SUV: Standardize Uptake Value, TLG: Total Lesion Glycolysis, MTV: Metabolic Tumor Volume, D_max_: Maximum distance between two lesions, CMR: Complete Metabolic Response, PMR: Partial Metabolic Response, SMD: Stable Metabolic Disease

**Fig. 4:**
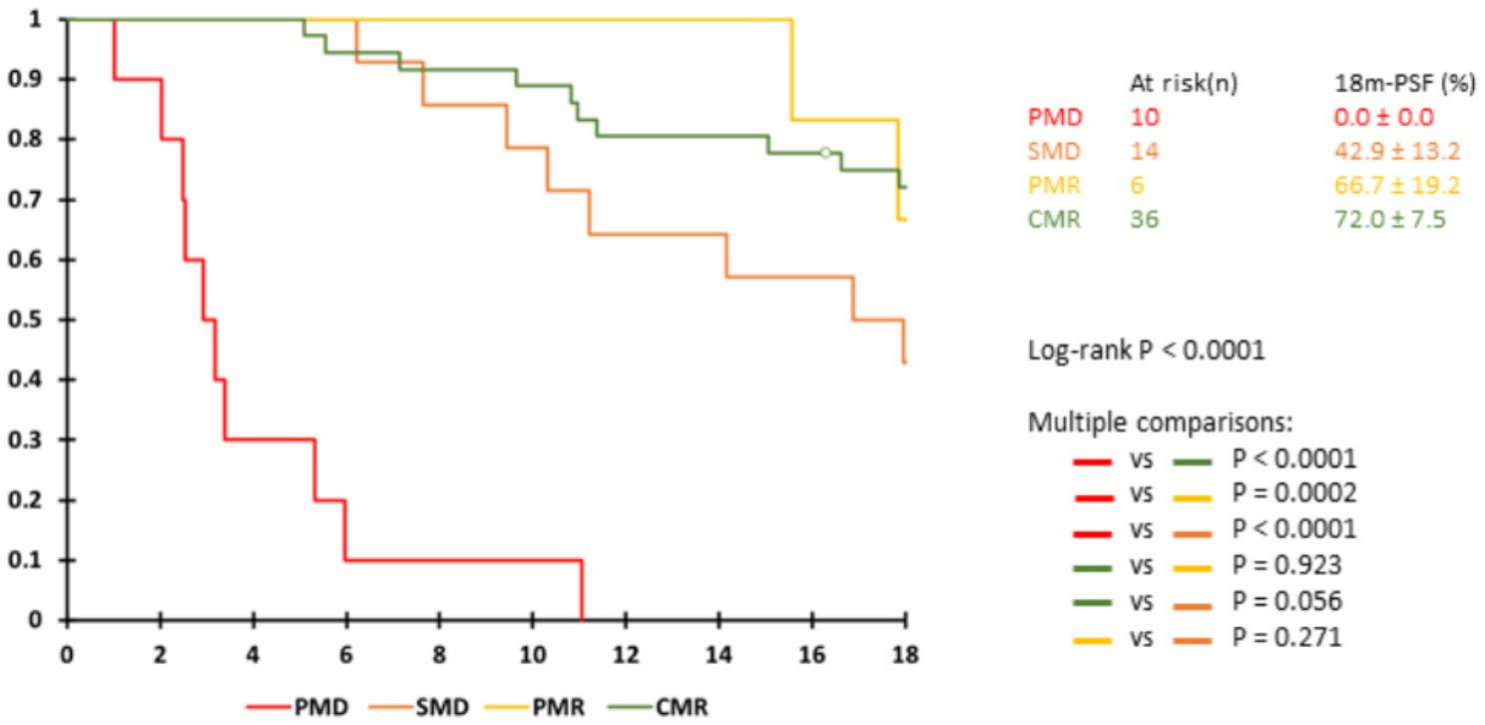
Kaplan-Meier analysis according to PERCIST classification at early PET evaluation

Regarding PERCIST response evaluation, there was no association between PERCIST classes and 18 m-PFS status (*p* = 0.149) and there was no difference in 18 m-PFS between patients classified as CMR, PMR and SMD (Fig. 4).

## Discussion

SUV and MTV at baseline have been shown to be good prognostic factors in a variety of cancers, including lung cancer [22], esophageal cancer [23], lymphoma [24] and head and neck carcinoma [25]. Despite considerable efforts to harmonize the measurement of these characteristics, the use of these metrics in interpretation and reporting continues to be inconsistent and controversial. However, high SUV_max_ and high MTV have been repeatedly shown to have negative prognostic value. More specifically in BC, recent systematic reviews and meta-analyses [26, 27] have also shown that patients with high SUV_max_, high MTV and high TLG had poorer prognosis. Although we used SUV_peak_ as per PERCIST recommendations instead of SUV_max_ in the present study, we did not find an association between baseline tumor metabolic intensity and/or volume and PFS at 18 months. As SUV was correlated to luminal type [27], this could be due to a selection bias. Indeed, the main studies exploring the prognostic value of SUV of the primary lesion in BC patients included various biological subgroups (i.e. luminal HER2-negative, HER2-positive and basal), whereas in the present study only luminal HER2-negative BC were concerned [28]. We assumed that in the specific group of luminal HER2-negative BC patients, tumor metabolism is not associated with the prognosis. The same likely applies to MTV and TLG. However, even if statistical significance was not reached for MTV and TLG, a trend coherent with previous findings could be observed, meaning that patients with higher baseline MTV and TLG had a trend towards poorer PFS.

However, since then, new quantitative PET parameters have been developed, including dissemination parameters such as D_max_, which corresponds to the maximal distance between the two farthest lesions in the examination. D_max_ has recently been explored quasi-exclusively in lymphomas [29] and some recent studies demonstrated in both Hodgkin lymphoma (HL) and non-HL lymphoma cohorts that combining D_max_ with texture features or clinical characteristics may lead to better predictive models compared to only MTV [21, 30]. We found similar results, as patients with a D_max_ < 18.10 cm had an 18-month PFS of 69.2%, compared with 36.7% for patients with a higher D_max_. Thus, at baseline, D_max_ appeared to be a more sensitive prognosticator than either SUV or MTV in patients with luminal HER2-negative BC patients, who are going to receive CdK4/6i in combination with ET. Moreover, the prognostic value of D_max_ does not depend on the metastatic disease location, patients with only bone metastatic disease displaying non-different D_max_ compared to others. This information could be used to adapt the therapeutic monitoring of patients according to the initial dissemination of the disease, either by spacing out the follow-up of patients with low dissemination or by reinforcing the follow-up of patients with high dissemination. However, the value of such strategies remains to be explored.

The prognostic utility of early PET therapeutic evaluation has been widely explored in many types of cancers and under different therapies with convincing results [31–35], and the possible clinical and financial implications need to be further explored. Here, early PET evaluation made it possible to depict 15% of early progression under CdK4/6i and thus allowed early treatment adaptation. However, the impact of such an early therapy switch remains to be explored. A recent retrospective study including 300 metastatic BC patients showed a survival benefit when ^18^F-FDG PET/CT was used alone or in combination with CT, with earlier detection of progressions of 5 months on average [36]. This indicated that detecting progression early and changing treatment may lead to a better chance of response to the subsequent treatment line. Of note, all but one of the early progressions were assessed on the appearance of new lesion(s) despite no significant increase in SUV_peak_. Furthermore, there was no difference in PFS at 18 months between CMR, PMR and SMD patients, which is in line with the absence of difference in ΔSUV_peak_, ΔMTV and ΔTLG between 18m-PFS0 and 18m-PFS1 responder patients. This finding suggests that in the absence of PMD on early metabolic progression, dual therapy with ET and CdK4/6i should be continued regardless of the quality of the initial response to treatment. As a previous study [37] showed no significant difference between the one- and five- lesion(s) PERCIST approaches for therapeutic evaluation of BC, we presently use the one-lesion approach for its simplicity, which makes it more easily transferable to routine clinical practice.

A notable limitation of this study is its retrospective and single-center design as well as the limited number of patients, although this is inherent to studies on new treatments and with a restricted selection of patients. In our institution, a complete PET follow-up including early therapeutic evaluation is not systematic. To avoid restricting the patient pool, patients from both first and second lines of metastatic treatment were included, along with both ductal and lobular subtypes. Consequently, this heterogeneity may potentially confound the results by introducing variability in treatment responses. Furthermore, another limitation arises from the lack of data concerning inter-observer variability, as the delineation of volumes was conducted only once. This absence of multiple assessments by different observers hinders the ability to assess the consistency and reliability of volume delineation, potentially impacting the accuracy and reproducibility of the findings.

## Conclusions

In ER + and HER2- mBC patients treated with CdK4/6i, disease dissemination at treatment initiation, evaluated by D_max_ on ^18^F-FDG PET/CT, emerges as a promising parameter for predicting event occurrence within the initial 18 months of treatment. Different strategies for incorporating this data into the therapeutic follow-up of patients remain to be evaluated. In addition, early ^18^F-FDG PET assessment at 3 months of treatment can detect up to 15% of early progression and is therefore highly encouraged to allow rapid therapeutic adjustment. Finally, in the absence of early metabolic progression, treatment should be continued regardless of the quality of the initial response to treatment.

## Data Availability

The datasets used and/or analysed during the current study are available from the corresponding author on reasonable request.

## Abbreviations

^18^F: FDG ^18^F-Fluorodeoxyglucose
FDG: Fluorodeoxyglucose
PET: Positron emission tomography
CT: Computed tomography
PFS: Progression free survival
ER: Estrogen receptor
HER2: Human epidermal growth factor receptor 2
MTV: Metabolic tumor volume
TLG: Total lesion glycolysis
D: _max_ Maximal distance
BC: Breast cancer
mBC: Metastatic breast cancer
ET: Endocrine Therapy
CdK4/6i: Cyclin-dependent kinase inhibitors
EUSOMA: European Society of Breast Cancer Specialists
VOI: Volume of interest
SUV: Standardized uptake value
CMR: Complete metabolic response
PMR: Partial metabolic response
PMD: Progressive metabolic disease
SMD: Stable metabolic disease
ROC: Receiver operator characteristic
AI: Aromatase inhibitor
